# Ustilago Maidis

**Published:** 1878-06-20

**Authors:** I. J. M. Goss

**Affiliations:** Marietta, Ga.


### USTILAGO MAIDIS.

By I. J. M. Goss, M.D., of Marietta, Ga.

(From his work on New Medicines.)

Ustilago, or corn-smut, is a fungus growing upon Indian corn; that upon the ear is best. The analysis of this fungus shows it to contain a large per cent. of ergotin, besides some other chemical constituents. The fluid extract and saturated tincture are forms in which I have mostly used the article.

*Medical Uses.*—Although it contains a large per cent. of ergotin, yet it acts somewhat differently from ergot, but it acts upon the impregnated uterus in a similar manner to ergot, but not so continuously—its action being remittent, like that of natural labor. It was first brought to the notice of physicians by its producing abortion upon cattle fed upon corn containing large quantities of this fungus. I have used it for several years in obstetrical practice, and find its action upon the uterus very positive, and, in most cases, more so than ergot. I had a case, recently, in which the labor was quite tardy, and had lingered until the lady was becoming exhausted, and I gave one-half drachm of the fluid extract, which immediately produced strong, expulsive contractions of the uterus, and terminated the labor in a few minutes, and that without any harm to the mother or child; this is its recommendation. I have given it frequently, and find that it acts as well, and better than ergot, and, at the same time, it does not produce toxical effects upon either mother or child. But, in many cases, where I had to continue ergot for some time, in cases of inertia of the uterus, I noticed that the infants were born dead, and in several cases, puerperal fever followed the free use of ergot. This result has never followed the use of ustilago in my hands, nor in the hands of others, that I have heard of. It does not produce that constant contraction that ergot produces—so exhausting to the female—but it produces remittent contractions, just like natural labor. These facts I have discovered by the frequent use of this article now for several years; and in cases of hemorrhage, either after labor or in the non-impregnated uterus, I find that ustilago is more active than ergot in arresting the hemorrhage. It so contracts the relaxed surface of the uterus as to close the vessels, and thereby check the flow. In passive hemorrhage, it may be given in small and repeated doses for several days without fear of any toxical effect, but with very passive results. The best preparation of it that I have ever found is a fluid extract prepared by Parke, Davis & Co., Detroit, Michigan.

